# Physician-Perceived Predictive Factors for the Effectiveness of Drugs for Treating Cancer Dyspnea: Results of a Nationwide Survey of Japanese Palliative Care Physicians

**DOI:** 10.1089/pmr.2020.0050

**Published:** 2020-06-30

**Authors:** Yoshinobu Matsuda, Ryo Matsunuma, Kozue Suzuki, Masanori Mori, Hiroaki Watanabe, Takashi Yamaguchi

**Affiliations:** ^1^ Department of Psychosomatic Internal Medicine, Kinki-Chuo Chest Medical Center, Sakai, Japan.; ^2^ Department of Palliative Medicine, Kobe University Graduate School of Medicine, Kobe, Japan.; ^3^ Department of Palliative Care, Tokyo Metropolitan Cancer and Infectious Disease Center Komagome Hospital, Tokyo, Japan.; ^4^ Palliative Care Team, Seirei Mikatahara General Hospital, Hamamatsu, Japan.; ^5^ Department of Palliative Care, Komaki City Hospital, Komaki, Japan.; ^6^ Division of Palliative Care, Department of Medicine, Konan Hospital, Kobe, Japan.

**Keywords:** benzodiazepine, cancer, corticosteroid, dyspnea, opioid

## Abstract

***Background:*** Dyspnea is a common and distressing symptom in patients with advanced cancer. Opioids, benzodiazepines, and corticosteroids are commonly prescribed pharmacological treatments for cancer dyspnea.

***Objective:*** The objective of this survey was to investigate physician-perceived predictive factors for the effectiveness of opioids, benzodiazepines, and corticosteroids in treating cancer dyspnea.

***Design:*** This study involves a nationwide survey using self-report questionnaires.

***Setting/Subjects:*** Random sampling selected 268 Japanese certified palliative care physicians in Japan.

***Measurements:*** We inquired about the 12 physician-perceived predictive factors for the effectiveness of drugs (opioids, benzodiazepines, and corticosteroids) in treating cancer dyspnea.

***Results:*** The frequently selected physician-perceived predictive factors for the effectiveness of opioids were tachypnea, respiratory effort, opioid naive, Eastern Cooperative Oncology Group Performance Status 0–2, multiple lung tumors, dry cough, pleural effusion, and pleural lesion. Benzodiazepines were predicted to be effective against dyspnea in patients with depression and severe anxiety. Meanwhile, corticosteroids were predicted to be effective against dyspnea in patients with lymphangitis carcinomatosa, superior vena cava syndrome, major airway obstruction, and audible wheezing. Japanese palliative care physicians anticipate that different drug classes will be effective for treating dyspnea in patients with specific factors.

***Conclusions:*** Japanese palliative care physicians expect that different drugs will be effective for dyspnea in patients with specific predictive factors. Future prospective studies are required to assess the effectiveness of each drug class against specific dyspnea.

## Introduction

Dyspnea is a common and distressing symptom in patients with advanced cancer.^1^ Opioids, benzodiazepines, and corticosteroids are commonly prescribed pharmacological treatments for cancer dyspnea. In the Japanese practice guidelines for the treatment of respiratory symptoms in patients with cancer, the following recommendations are made for the treatment of dyspnea: systemic morphine is recommended; systemic oxycodone is suggested; benzodiazepines are suggested, but only in combination with opioids and not alone; and systemic corticosteroids are not suggested for routine use without consideration of dyspnea etiology, whereas they are suggested for patients with lymphangitis carcinomatosa, superior vena cava (SVC) syndrome, or major airway obstruction.^2^

Cancer dyspnea includes dyspnea caused by variety of conditions. As already mentioned, different pharmacological treatment options are suggested for specific cancer dyspnea. However, the factors predicting the effectiveness of pharmacological treatment of cancer dyspnea have not yet been fully revealed. As a first step toward treatment standardization of drugs for each specific cancer dyspnea, understanding the current practices of palliative care physicians is important. Thus, we conducted a survey among palliative care physicians to investigate physician-perceived predictive factors for the effectiveness of opioids, benzodiazepines, and corticosteroids in the treatment of cancer dyspnea.

## Methods

A nationwide survey of Japanese-certified palliative care physicians was conducted using self-report questionnaires. This study was approved by the institutional review board of Konan Hospital.

### Subjects

The Japanese Society for Palliative Medicine website was used to identify a total of 536 certified palliative care physicians as potential participants. Questionnaires were distributed to 268 (50%) physicians randomly selected from those identified, and the selected physicians were divided into two groups. One group received a questionnaire regarding the management of dyspnea in ambulatory cancer patients, and the other received a questionnaire regarding the management of dyspnea in cancer patients at the end of life. The use of two groups shortened the length of the questionnaires and minimized the burden on the physicians. The data presented in this article were obtained from the questionnaire regarding the management of dyspnea in ambulatory cancer patients. The results for the other topics on the survey, including the drug classes and dosages, have been presented elsewhere.^3–7^ In this study, we focus on exploring the factors predicting the effectiveness of pharmacological treatment of cancer dyspnea.

### Procedure

The self-report questionnaire was distributed by mail to the selected potential participants in September 2018.

### Measurements

As no preexisting questionnaires matched our specific requirements, we developed a novel *ad hoc* questionnaire based on a literature review and discussion among the investigators.^1,2,8–16^ We described a hypothetical scenario of an adult ambulatory cancer patient with an expected survival of more than several months and a dyspnea score of 2 or 3 on the modified Medical Research Council Scale. For this hypothetical case, we inquired about the physician-perceived predictive factors for the effectiveness of drugs (opioids, benzodiazepines, and corticosteroids) in treating cancer dyspnea.

The following predictive factor options were provided: (1) Eastern Cooperative Oncology Group Performance Status (ECOG PS) 0–2; (2) tachypnea (i.e., respiratory rate ≥24/min); (3) SpO_2_ <90%; (4) audible wheezing; (5) dry cough; (6) productive cough; (7) respiratory effort; (8) air hunger; (9) chest tightness; (10) severe anxiety; (11) depression; (12) primary or metastatic multiple lung tumors; (13) pleural lesions that limit thoracic movement; (14) pleural effusion, that is, more than half of unilateral thoracic cage; (15) major airway obstruction, that is, trachea or main bronchus; (16) SVC syndrome; (17) lymphangitis carcinomatosa; and (18) opioid naive.

### Statistical analysis

All data were managed and analyzed using SPSS, version 23.0. The proportion of participants responding that they expected the effectiveness of each drug given each predictive factor and 95% confidence intervals (CIs) were calculated.

## Results

The questionnaires were returned by 192 physicians, providing a response rate of 71.6%. All responses were included in the subsequent analysis.

### Opioids

A high proportion of physician participants reported expecting opioids to be effective for the treatment of dyspnea in patients with tachypnea (89.6%, 95% CI: 84–93), respiratory effort (82.3%, 95% CI: 76–87), opioid naive (82.3%, 95% CI: 76–87), ECOG PS 0–2 (81.3%, 95% CI: 75–86), primary or metastatic multiple lung tumors (75.0%, 95% CI: 68–81), dry cough (72.9%, 95% CI: 66–79), pleural effusion (71.9%, 95% CI: 65–78), and pleural lesions (70.8%, 95% CI: 64–77; Fig. 1).

**FIG. 1.**
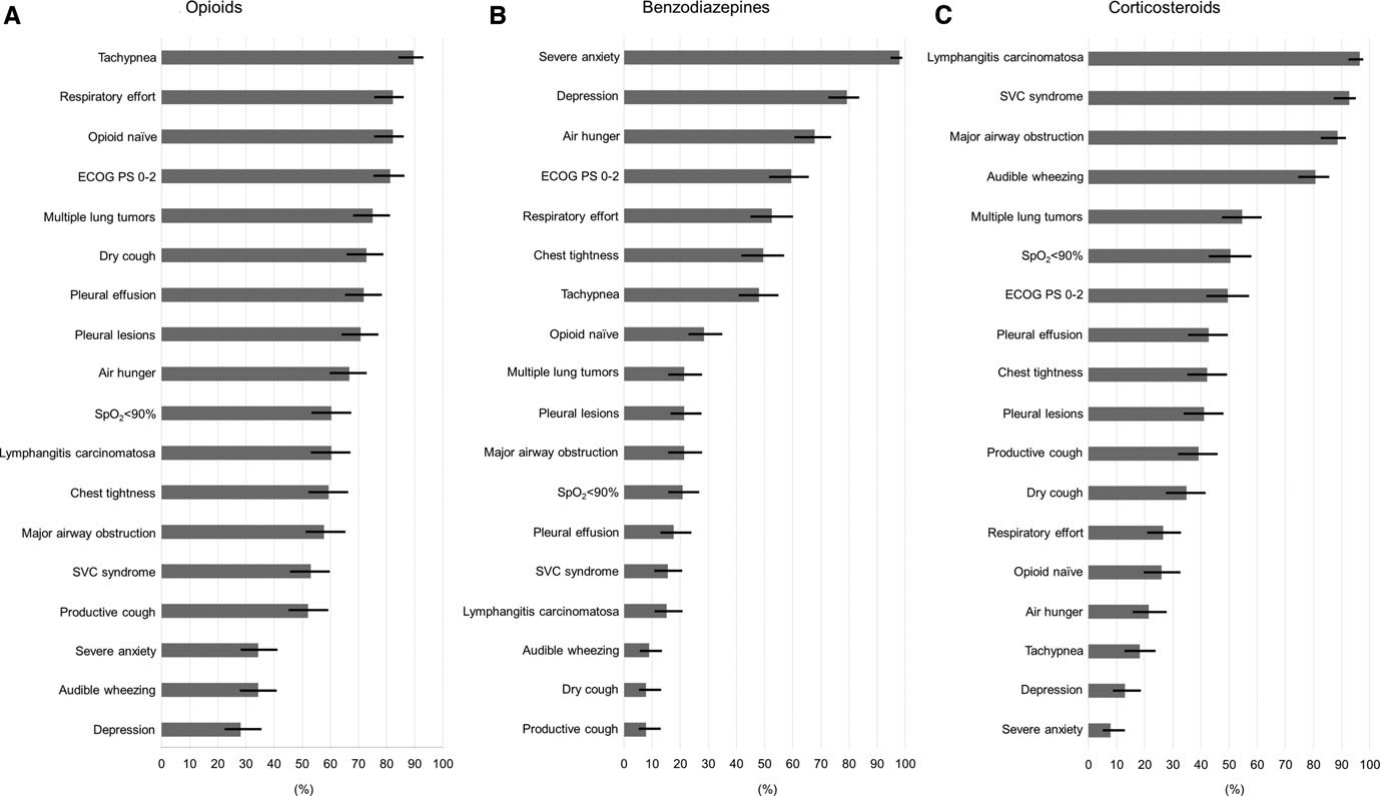
Physician-perceived predictive factors for the effectiveness of drugs (*n* = 192). Error bars indicate 95% confidence intervals. SVC, superior vena cava.

### Benzodiazepines

The proportion of participants who reported expecting benzodiazepines to be effective for treating dyspnea was high for patients with severe anxiety (97.9%, 95% CI: 95–99) and depression (79.2%, 95% CI: 73–84; Fig. 1).

### Corticosteroids

The proportion of participants who reported expected corticosteroids to be effective was high for patients with lymphangitis carcinomatosa (96.4% 95% CI: 93–98), SVC syndrome (92.7%, 95% CI: 88–96), major airway obstruction (88.5% 95% CI: 83–92), and audible wheezing (80.7%, 95% CI: 75–86; Fig. 1).

## Discussion

To the best of our knowledge, this is the first nationwide survey of palliative care physicians investigating physician-perceived predictive factors for the effectiveness of opioids, benzodiazepines, and corticosteroids for the treatment of dyspnea in patients with cancer in Japan.

The first finding of this survey was that participants expect opioids to effectively treat dyspnea in patients with tachypnea, respiratory effort, opioid naive, ECOG PS 0–2, primary or metastatic multiple lung tumors, dry cough, pleural effusion, and pleural lesions. Relatively more factors were raised as physician-perceived predictive factors of the effectiveness of opioids compared with factors for benzodiazepines or corticosteroids. This can be explained by the fact that opioids, especially morphine, are recommended as the first line of pharmacological treatment for dyspnea in patients with cancer according to some guidelines.^1,6^ In addition, other effects of opioids may influence these results, that is, a possible reduction of respiratory rate improving tachypnea or respiratory effort, and an antitussive effect against dry cough.^16–18^ However, a previous study has shown that opioid treatment neither causes a decrease in respiratory rate nor the effectiveness of opioids for treating patients with tachypnea.^19^ Good PS may be selected for because dyspnea worsens in the terminal phase, when PS also declines.^20^ It will be important to reveal the predictive factors for the effectiveness of opioids in the real world. Therefore, we are currently conducting a prospective study of opioids for cancer dyspnea.^21^

The second finding was that the participating physicians did not expect opioids to be effective for treating dyspnea in patients with depression and severe anxiety, but they did expect benzodiazepines to be effective. As already mentioned, opioids are the first-line pharmacological treatment for cancer dyspnea; however, their efficacy is minimal. Opioids provide reduction on the numerical rating scale (NRS) of only ∼1 point.^22^ Physicians may regard dyspnea in these patients as total dyspnea, that is, including psychological and social factors along with physical symptoms, and try to treat it comprehensively. Therefore, they may prefer to treat patients with benzodiazepines over opioids, as benzodiazepines additionally treat severe anxiety and depression.

Dyspnea in cancer patients is associated with anxiety.^23–25^ In our previous report, palliative care physicians reported that they prescribe benzodiazepines to patients with anxiety more frequently than to those without anxiety.^7^ The results of this survey regarding benzodiazepines are in line with this result. A randomized controlled trial was previously conducted on cancer patients with dyspnea comparing morphine alone, midazolam alone, or morphine and midazolam given together; patients given both morphine and midazolam reported relief of dyspnea at a significantly higher frequency than those in the group given morphine alone.^26^ However, a systematic review concluded that there is no evidence for or against the use of benzodiazepines for the relief of dyspnea.^27^ Therefore, further studies are needed to investigate the efficacy of benzodiazepines for cancer dyspnea in patients with anxiety.

Almost 80% of physicians reported depression to be a predictive factor for the effectiveness of benzodiazepines. Depression is associated with dyspnea and is a strong predictor of anxiety in patients with cancer.^23,28^ Physicians, therefore, may use depression as a predictive factor for the effectiveness of benzodiazepines. However, benzodiazepines are not mentioned in the guidelines for the management of depression in patients with cancer.^29^ Instead, these guidelines recommended antidepressants for the treatment of depression. A randomized controlled trial of sertraline in patients with chronic breathlessness failed to provide any benefits in symptom relief over a placebo.^30^ Currently, a randomized control trial assessing mirtazapine for the treatment of dyspnea in patients with advanced lung disease is ongoing.^31^ We did not include antidepressants as a treatment for dyspnea in this survey as this survey was designed before the results of the feasibility study on mirtazapine were published.^31^ Future studies are needed to assess the effectiveness of benzodiazepines or antidepressants for the treatment of cancer dyspnea in patients with depression.

The third finding is that participants expected corticosteroids to be effective in treating dyspnea in patients with lymphangitis carcinomatosa, SVC syndrome, major airway obstruction, and audible wheezing. These results are in line with the recommendations in the guidelines for the treatment of dyspnea in patients with cancer.^2^ Hui et al. conducted a pilot double-blind randomized controlled trial of dexamethasone for dyspnea in cancer patients.^9^ They reported that dexamethasone was associated with a significant reduction in dyspnea due to lung involvement, including lymphangitis carcinomatosa. The results of their study suggest that corticosteroids may be effective for cancer dyspnea in patients with these predictive factors.

Participants might have selected audible wheezing as a physician-perceived predictive factor of corticosteroid effectiveness because corticosteroids are often used for wheezing in bronchial asthma attacks or in exacerbation of chronic obstructive pulmonary disease as well as lymphangitis carcinomatosa. In addition, Mori et al. conducted a prospective observational study to explore potential factors predicting cancer patients' response to corticosteroids for dyspnea.^8^ They reported that some factors, including the presence of audible wheezing, are associated with the response to corticosteroids, although their study was a preliminary study. Therefore, larger prospective studies in patients including those with relatively rare conditions, such as lymphangitis carcinomatosa, SVC syndrome, and major airway obstruction, are required.

The strengths of this study are that the sample was drawn from the registry of certified palliative care physicians of the Japanese Society of Palliative Medicine and a high response rate (>70%) was obtained. However, this study has some limitations. First, this was a survey of physician-reported practice; thus, there may be discrepancies between the self-reported data and actual clinical practices. Second, we did not ask for the background information of participants to minimize the burden imposed on them while responding to the questionnaire, which may have contributed to obtaining a higher response rate. Third, we did not request qualitative data from the participating physicians. To explore the rationale behind their reports, future qualitative surveys are needed. Finally, we did not investigate physician-perceived predictive factors for the effectiveness of antidepressants, which may also be revealed by further surveys.

## Conclusions

Japanese palliative care physicians expect that different drugs will be effective for dyspnea in patients with specific predictive factors. Future prospective studies are required to assess the efficacy of each drug against dyspnea in cancer patients with each factor.

## Abbreviations Used

CI: confidence interval
ECOG PS: Eastern Cooperative Oncology Group Performance Status
SVC: superior vena cava
